# Amelioration of Cadmium-Induced Oxidative Damage in Wistar Rats by Vitamin C, Zinc and *N*-Acetylcysteine

**DOI:** 10.3390/medsci10010007

**Published:** 2022-01-26

**Authors:** Venkataramanaiah Poli, Renuka Madduru, Yenukolu Aparna, Vimala Kandukuri, Srinivasulu Reddy Motireddy

**Affiliations:** Department of Zoology, Sri Venkateswara University, Tirupati 517502, India; ramanasvu10@gmail.com (V.P.); renumdru@gmail.com (R.M.); ramaparn@gmail.com (Y.A.); chinnikandukuri444@gmail.com (V.K.)

**Keywords:** cadmium, vitamin C, zinc, *N*-acetylcysteine, amelioration potential

## Abstract

The present study was performed to determine the protective effects of vitamin C, zinc, and *N*-acetylcysteine, individually or in combination with Cd, to monitor their amelioration capability against Cd-induced oxidative damage in Wistar rats. We investigated and demonstrated that cadmium is a toxic element that damages rat liver and kidney tissues. Vitamin C, zinc, and NAC have been proven to have protective roles against Cd toxic effects. Nine groups of rats were studied as the experimental group. The present experiment was conducted for 45 days. Liver and kidneys were excised for biochemical evaluation by assaying antioxidant enzymes and lipid oxidation products to assess the impact of Cd toxicity and its amelioration by co-administration of vitamin C, zinc, and NAC along with Cd. Basal metabolic rates and tissue respiration rates of liver and kidney were significantly decreased (*p* < 0.05) during Cd toxicity. Serum biochemical parameters were also found to be significantly altered to cope with Cd toxicity. All the antioxidant enzymes and products were significant inhibited (*p* < 0.05) or elevated in rat liver and kidney tissues during Cd-induced toxicity. Our results suggest that co-administration of vitamin C, zinc, and NAC to rats ameliorates oxidative damage induced by Cd toxicity. From the results obtained in the present study, all the agents tested had protective effects against Cd-induced oxidative damage.

## 1. Introduction

Metallic elements are intrinsic components of the environment which are difficult to remove completely. With increasing use of a wide variety of metals in industry and in our daily life, serious problems resulting from toxic metal pollution of the environment have been reported [1,2]. In recent years, there have been increasing ecological and global public health concerns associated with environmental contamination by these metals. Furthermore, human exposure has risen dramatically as a result of an exponential increase of their use in several industrial, agricultural, domestic and technological applications [2]. Heavy metals can be accumulated by organisms through a variety of pathways, including respiration, absorption and ingestion, with high toxicity to many organs of both humans and animals [3,4]. Cadmium (Cd) is one of the most toxic heavy metals. It is used in the manufacture of pigments, stabilizers, alloys, and electronic compounds, and, in particular, in rechargeable nickel-cadmium batteries. Thus, its presence in the environment has increased with industrial development [5,6].

Cd as a naturally occurring metal can be found in food, water, and cigarette smoke. However, the mechanisms and pathways for the toxic effects of Cd at tissue level are not yet well understood in biological systems. Several studies have shown that Cd toxicity stimulates the production of reactive oxygen species (ROS) and the induction of oxidative stress in different organs [7]. Moreover, Cd exposure stimulates lipid-peroxidation-induced tissue damage [8]. In living cells, protection against oxidative damage encompasses enzymatic and non-enzymatic antioxidant systems. Cd induces oxidative stress through impairment of the antioxidant enzyme system due to changes in gene expression mechanisms [9]. Disruption of the cellular oxido-reduction balance may lead to severe damage, at both tissue and organ levels, leading to impaired function [5]. Currently, researchers are working on several issues relating to antioxidant status under Cd intoxication in several systems. It has been well established that endogenous antioxidants play a pivotal role in antioxidant defense mechanisms against oxidative impairment, reflecting the protective role of particular biological functions [7,10].

Natural antioxidants, vitamins A, D, E, carotenoids, and certain elements, which are consumed in the diet, play a vital role in the maintenance of antioxidant status in biological systems. Recently, much interest has been shown in herbal antioxidants as preventatives in opposition to oxidative damage in the pathophysiology of various health issues [11]. Among a range of antioxidants, vitamins, zinc and *N*-acetylcysteine (NAC) were found to play an important role in the regulation of the antioxidant system [7,10]. Vitamin C belongs to the category of water soluble, chain-breaking, antioxidant vitamins and is capable of scavenging both superoxide and hydrogen peroxide [12]. Zinc is considered one of the most important essential trace elements for human beings and forms an integral component of several enzymes involved in catalytic activity [10]. NAC is a relatively small molecule containing a thiol group and can perform antioxidant defense functions. It is active in all intracellular components. The pharmacological applications and properties of NAC are assumed to be due to the presence of the thiol group, which can reduce other thiol groups to scavenge free oxygen molecules. A large amount of information on the effect of environmental xenobiotics is available. However, the impact of Cd, either individually or in combination with available natural antioxidants and trace elements, requires to be elucidated in the case of biological systems, which would facilitate understanding of the interaction of Cd with available antioxidants.

Therefore, the present investigation sought to address the impact of oxidative and antioxidant defense mechanisms at a cellular level and to examine whether the administration of vitamin C, zinc, and NAC, both individually and in combination, can reverse Cd-induced tissue oxidative damage or affect biochemical pathways, using oxidative marker enzymes as oxidative defense biomarkers.

## 2. Materials and Methods

### 2.1. Animals

Male rats (225 ± 10 g) were selected and kept under laboratory (temperature 23 ± 20 °C; relative humidity 50 ± 10%, L:D cycle 12:12) conditions and kept in stainless steel mesh cages. The rats were fed ad libitum with rat chow (obtained from M/S Sai Durga Feeds, Bangalore) and provided with a drinking water facility and acclimatized to laboratory conditions for 10 days. The Institutional Animal Ethics Committee approved the experimental protocols, and animal use (Resolution No. 60b/2012/(i)/a/CPCSEA/IAEC/SVU/MSR–RS dt. 08.07.2012), Sri Venkateswara University, Tirupati, Andhra Pradesh, India.

### 2.2. Chemicals

Cadmium as cadmium chloride (CdCl_2_), zinc as zinc chloride (ZnCl_2_), and vitamin C (ascorbic acid) were obtained from Sigma Chemical Co., Loba Chemicals and Sd-Fine chemicals, India. All the chemicals used in this study were of the highest purity.

### 2.3. Experimental Design

Rats were divided into 9 batches; each comprising of 6 rats and fed one of the following diets.

Batch1: Control batch

Batch2: CdCl_2_ dissolved in drinking water at 10 mg/L

Batch3: CdCl_2_ (10 mg/L of drinking water) + vitamin C (100 mg/kg BW)

Batch 4: CdCl_2_ (10 mg/L of drinking water) + zinc (15 mg/kg)

Batch 5: CdCl_2_ (10 mg/L of drinking water) + NAC (100 mg/kg through gavage)

Batch 6: CdCl_2_ (10 mg/L drinking water) + vitamin C (100 mg/kg BW) + zinc (15 mg/kg)

Batch 7: CdCl_2_ (10 mg/L drinking water) + vitamin C (100 mg/kg BW) + NAC (100 mg/kg through gavage)

Batch 8: CdCl_2_ (10 mg/L drinking water) + zinc (15 mg/kg) + NAC (100 mg/kg through gavage)

Batch 9: CdCl_2_ (10 mg/L drinking water) + vitamin C (100 mg/kg BW) + zinc (15 mg/kg) + NAC (100 mg/kg through gavage)

The quantity of food consumption and drinking water were monitored and found to be 35–60 g forage/day and 25–40 mL/day per rat, respectively.

After completing the study, all the animals were accurately weighed and the weights were recorded. The animals were anaesthetized, and blood samples were collected through cardiac punctures. The animals were sacrificed by cervical dislocation, and the tissues, such as the liver and kidney, were isolated and kept at 4 °C for further biochemical analysis. Serum samples were separated using centrifugation at 2000 rpm for 20 min and used for biochemical analysis.

### 2.4. Biochemical Analyses Were Performed by the following Methods

The organ weights were presented as relative organ weights calculated as follows:

$$\mathrm{Relative}\;\mathrm{organ}\;\mathrm{weight}:\;\frac{\mathrm{Absolute}\;\mathrm{organ}\;\mathrm{weight}\;\left(g\right)}{\;\mathrm{Whole}\;\mathrm{body}\;\mathrm{weight}\;\left(g\right)}\times 100$$

#### 2.4.1. Tissue Respiration

The rate of oxygen consumption in the liver and kidney tissues of control and Cd-intoxicated rats were measured using a Warburg constant volume respirometer (Biochem, India) by the procedure described by Umbriet et al., (1959) [13]. The manometers and flasks were calibrated with mercury. Brodie’s fluid was used as manometric fluid. The Warburg’s flasks were cleaned with chromosulfuric acid, washed thoroughly and dried in a hot air oven before use. Mammalian ringer prepared in glass distilled water was used as the tissue suspension medium.

The mammalian ringer had the following composition (2.2 mM CaCl_2_, 5.5 mM KCl, 154 mM NaCl, 2.4 mM NaHCO_3_, 2 mM Tris-Cl pH 8.0). The pH was adjusted to 7.4 with HCl.

The isolated liver and kidney tissues were placed in microbeakers containing cold mammalian ringer solution for some time to recover from shock effects. After accurately weighing the tissues in an electrical balance, the tissues were cut into thin slices and teased. The processed tissues were transferred to individual Warburg flasks containing 3 mL of ringer solution. A drop of penicillin was also added to each of the tissue preparations to prevent bacterial growth. A quantity of 0.2 mL of 20% potassium hydroxide (KOH) solution and a filter paper wick were introduced into the central well of each flask for carbon dioxide absorption. Finally, the flasks were mounted on to the manometers. The entire setup was allowed to equilibrate for approximately 15 min. Thus, in each manometer, the gas phase was the air and the liquid phase was the ringer solution. After ensuring the setup was air-tight, the oxygen consumption of the different tissues was measured at 10 min intervals.

The rate of tissue respiration was calculated as per the procedure given by Umbriet et al., (1959) as:

$${\mu L\;\mathrm{of}\;O}_{2}\;\mathrm{consumed}=\frac{h\;\times \;K}{\;\mathrm{Wt}.\;\mathrm{of}\;\mathrm{the}\;\mathrm{tissue}\;\mathrm{taken}}\times 1000$$

where h = The observed change in manometer

K = flask constant

$$\mathrm{Derivation}\;\mathrm{of}\;K=\frac{\mathrm{Vg}\;\frac{273}{T}{+\mathrm{Vf}}_{\alpha }}{\;\mathrm{Po}}$$

where Vg = volume of gas phase in flask including tubes down to the reference point.

Vf = volume of fluid in vessel

Po = standard pressure which is 760 mm Hg or 10,000 mm Brodie’s units

T = temperature of water bath in absolute degree (273 + 37 °C)

A = solubility in reaction liquid of gas involved (expressed as mL gas/mL liquid) when gas is at a pressure 760 mm Hg at temperature T (0.0239).

The values were expressed as µL of O_2_/g wet weight tissue/h.

#### 2.4.2. Total Protein

Total protein in the tissues and serum were estimated using Lowry’s method (Lowry et al., 1954) [14]. Quantities of 10 mg containing tissue homogenates were taken and precipitated using 1 mL TCA (10%) and then centrifuged at 5000× *g* for 10 min. The supernatant was discarded and the residue was dissolved in 0.5 mL of NaOH (0.1 N). An amount of 0.1 mL of the dissolved protein residue was taken and 5 mL of alkaline copper sulphate was added. The mixture was incubated for 5 min followed by addition of 1 N Follin’s reagent and further incubated for 10 min in the dark. Readings were taken at 620 nm using a U-2900 UV-VIS double beam spectrophotometer (High Technologies, Santa Clara, CA, USA) against the reagent blank, using bovine serum albumin as standard.

#### 2.4.3. Urea

Urea content was estimated by the method of diacetyl monoxime, as described by Natelson (1971) [15]. Serum samples were collected and 15% PCA to 0.5 mL of supernatant, 1 mL of acid mixture (1:3 sulfuric acid: phosphoric acid) and 0.5 mL of 2% diacetylmonoxime were added and vortexed. The contents were boiled in a boiling water bath for 30 min and the contents were then immediately cooled to laboratory temperature. Then the samples were read at 480 nm against the reagent blank in a spectrophotometer. Urea content was expressed as µ moles of urea/gm wet weight of tissue.

#### 2.4.4. Creatinine

Blood samples were collected at the end of the exposure period into plain containers and separated into serum for the analysis. Serum creatinine estimation was carried out on the serum sample collected from both the control and the test male Wistar rats using the urease kinetic method and Jaffe’s reaction method, respectively, with a Roche/Hitachi Cobas C 311 auto-analyzer (Hawk and Loser, 1948) [16].

#### 2.4.5. Lactate Dehydrogenase (LDH)

Lactate dehydrogenase activity was determined by the method described by Nachlas et al., (1960) [17] with slight modifications. The serum samples were centrifuged at 1000× *g* for 15 min at 4 °C. The supernatant fraction was used for enzyme assay. The reaction mixture had a final volume of 2 mL containing 40 μ moles of sodium lactate, 100 μ moles of sodium phosphate buffer (pH 7.4), 0.1 μ mole of NAD and 4 μ moles of INT. The reaction was initiated by the addition of 0.2 mL of homogenate containing 20 mg of tissue as an enzyme source and incubated for 30 min at 37 °C. The reaction was stopped by the addition of 5 mL of glacial acetic acid. Zero-time controls (ZTCs) were maintained by addition of 5 mL of glacial acetic acid prior to the addition of the enzyme source to the incubation mixture. The formazan formed was extracted overnight into 5 mL of toluene at 5 °C. The color developed was measured at 495 nm in a spectrophotometer against the toluene blank. The enzyme activity was expressed in μ moles of formazan formed/mg protein/hour.

#### 2.4.6. Aspartate Amino Transferase (AST)

The activity of AST was assayed by the colorimetric method of Reitman and Frankel (1957) [18] as described by Bergmeyer and Bernt (1965). The serum samples were centrifuged at 1000× *g* for 15 min and supernatant was used for the enzyme assay. The incubation mixture of 2.0 mL contained 100 μ moles of phosphate buffer (Na_2_HPO_4_ + NaH_2_PO_4_) (pH 7.4), 100 μ moles of L-aspartic acid, 2 μ moles of α-keto glutamate and 0.5 mL of supernatant as enzyme source. After incubation for 30 min at 37 °C, the reaction was stopped by the addition of 1 mL of ketone reagent (0.001 M, 2,4-dinitrophenyl hydrazine solution in 1 N HCl) and the contents were allowed to stay at laboratory temperature for 20 min. After 20 min, 10 mL of 0.4 N NaOH was added. The developed color was read at 545 nm in a spectrophotometer against a reagent blank. The enzyme activity was expressed as μ moles of pyruvate formed/mg protein/hour.

#### 2.4.7. Alanine Amino Transferase (ALT)

The serum ALT activity was assayed by the colorimetric method of Reitman and Frankel (1957) [18] as described by Berg Meyer and Bernt (1965). The incubation mixture of 2 mL contained 100 μ moles of DL-alanine, 100 μ moles of phosphate buffer (pH 7.4), 2 μ moles of α-ketoglutarate and 0.5 mL of the supernatant of the homogenate. A quantity of 2% *w*/*v* was prepared in 0.25 M ice-cold sucrose solution, as enzyme source. The reaction mixture was incubated at 37 °C for 30 min. The reaction was stopped by the addition of 1.0 mL of 2,4-dinitrophenyl hydrazine solution prepared in 1 N HCl (ketone reagent). The color was developed by the addition of NaOH, as described above for ALT, and then measured at 545 nm in a spectrophotometer against the reagent blank. The enzyme activity was expressed as μ moles of pyruvate formed/mg/protein/hour.

#### 2.4.8. Malondialdehyde (MDA)

MDA concentration in tissues such as the liver and kidney was measured using the wavelengths of 552 nm for emission and 515 nm for excitation using a Perkin Elmer LS45 spectrofluorimeter, (PerkinElmer, Inc., Waltham, MA, USA) by Ohkawa et al., (1979) [19] and standard curve prepared for 1,1,3,3-tetraethoxypropane, the product of malondialdehyde and thiobarbituric acid reaction. MDA concentration was expressed in µmol/g protein.

#### 2.4.9. Superoxide Dismutase (SOD)

Superoxide dismutase activity was determined according to the method of Misra and Fridovich, (1972) [20] at room temperature. The isolated experimental tissues were homogenized in ice cold phosphate buffer (50 mM; pH 7.0), containing 0.1 mM EDTA to arrive at desired homogenate (5% *w*/*v*). The prepared homogenates were subjected to cold centrifugation at 10,000 rpm for 10 min at 4 °C. After the centrifugation, the supernatant was used for further enzyme assays. A quantity of 100 μL of tissue extract was added to 880 μL (0.05 M, pH 10.2, containing 0.1 mM EDTA) carbonate buffer and 20 μL of 30 mM epinephrine (in 0.05% acetic acid) was added to the mixture and the optical density was measured at a value of 480 nm for 4 min using a Hitachi U-2000 spectrophotometer. Activity was expressed as the amount of enzyme that inhibited the oxidation of epinephrine by 50%, which is equal to 1 unit.

#### 2.4.10. Catalase (CAT)

Catalase activity was measured by a slightly modified version of Aebi (1984) [21]. The desired concentrations (*w*/*v*) of tissue homogenates were prepared in ice cold 50 mM phosphate buffer (pH 7.0) containing 0.1 mM EDTA and then subjected to centrifugation at 10,000 rpm for 10 min at 4 °C. The supernatant obtained was used for enzyme assay. A quantity of 10 μL of 100% ethanol was added to 100 μL of tissue extract and then placed in an ice bath for 30 min. After 30 min the tubes were kept at room temperature followed by the addition of 10 μL of Triton X-100 RS. To a cuvette containing 200 μL of phosphate buffer and 50 μL of tissue extract was added 250 μL of 0.066 M H_2_O_2_ (in phosphate buffer) and decreases in optical density measured at 240 nm for 60 s in a UV spectrophotometer. A molar extinction coefficient of 43.6 M cm^−1^ was used to determine CAT activity. The unit of activity is equal to moles of H_2_O_2_ degraded/mg protein/min.

#### 2.4.11. Lipid Peroxidation (LPO)

The assay used for MDA levels was used as described by the method of Okhaw et al. (1979) [19]. The tissues were homogenized (5% *w*/*v*) in 50 mM phosphate buffer (pH 7.0) with 0.1 mM EDTA, then homogenates were centrifuged at 10,000 rpm for 10 min at 4 °C in cold centrifuge. The separated supernatant part was used for the estimation. A quantity of 200 μL of the tissue extract was added to 50 μL of 8.1% sodium dodecyl sulphate (SDS) and vortexed and incubated for 10 min at room temperature. A quantity of 375 μL of 20% acetic acid (pH 3.5) and 375 μL of thiobarbutiric acid (0.6%) were added and placed in a boiling water bath for 1 h. The samples were allowed to cool at room temperature. A mixture of 1.25 mL of butanol: pyridine (15:1) was added, vortexed and centrifuged at 1000 rpm for 5 min. The colored layer (500 μL) was measured at 532 nm using 1,1,3,3-tetraethoxy propane as a standard. The values were expressed in μ moles of malondialdehyde formed/g. wet wt of tissue or mL/minute.

#### 2.4.12. Glutathione Peroxidase (GPx)

The activity of glutathione peroxidase was analyzed by a modified Mills method (Mills, 1959) [22] as described by Hafeman et al. (1974). Glutathione peroxidase degrades hydrogen peroxide in the presence of glutathione thereby depleting it. The remaining glutathione (GSH) was then measured using 5, 51-dithiobis 2-nitrobenzoic acid (DTNB). The incubation mixture at 37 °C contained 80 mm sodium sulphate buffer (pH 7.0), 80 mm EDTA, 1 mm sodium azide, 0.4 mm glutathione, 0.25 mm hydrogen peroxide and tissue homogenate. After 3 min, aliquots of this solution were removed and treated with metaphosphoric acid precipitation solution. The glutathione in the protein-free filtrate was then determined using 0.4 m disodium hydrogen phosphate and 1 mm DTNB in 1% trisodium citrate solution. The absorbance of this solution was recorded at 412 nm in a spectrophotometer. A blank was carried out through the incubation simultaneously with the samples, since non-enzymatic glutathione oxidation by H_2_O_2_ occurs during incubation. One unit of glutathione peroxidase enzyme activity was defined as 1 µg of glutathione consumed per minute. The enzyme activity was expressed as g glutathione depleted per minute per milligram protein.

#### 2.4.13. Glutathione (GSH)

Glutathione content was determined according to the method of Theodorus et.al., (1981) [23]. The lung tissue was homogenized in 0.1 M ice cold phosphate buffer (pH 7.0) containing 0.001 M EDTA and protein was precipitated with 1 mL of 5% sulfosalicylic acid (*w*/*v*) and the contents were centrifuged at 5000× *g* for 15 min at 4 °C. The resulting supernatant was used as the enzyme source. The reaction mixture in a total volume of 2.5 mL contained 2.0 mL of 0.1 M potassium phosphate buffer, 0.05 mL of NADPH (4 mg/mL of 0.5% NaHCO_3_), 0.02 mL of DTNB (1.5 mg/mL), 0.02 mL of glutathione reductase (6 units/mL) and required amount of tissue source. The reaction was initiated by adding 0.41 mL of enzyme source and change in absorbance was recorded at 425 nm against the reagent blank in a spectrophotometer. The glutathione content was expressed in nanomoles/gram wet weight of the tissue.

### 2.5. Statistical Analysis and Data Presentation

The results obtained were presented as mean ± SD for comparison of different experimental animal batches with control batches. All the data obtained were statistically analyzed using the SPSS package. The results were statistically analyzed by one-way ANOVA; *p* value < 0.05 was considered significant. The biochemical measurement data were further subjected to an estimation of percent changes, caused by exposure to Cd with co-administration with vitamin C, zinc, and NAC individually and in combination.

## 3. Results

In the present study, the experimental period was fixed for 45 days, and the nine batches of rats were maintained accordingly. During the experimental periods, no mortalities were recorded.

### 3.1. Body Weights and Relative Tissue Weights

Initial body weight (IBW), final body weight (FBW) and relative tissue weight for liver and kidney of male rats from different experimental batches were recorded and presented in Table 1. The FBWs were significantly decreased (−45.89%; *p* < 0.05) compared to control, whereas for Cd-cotreatment, individually with vitamin C, zinc or NAC or in combination, recorded FBWs were not significant. Similarly, the body weight gain or loss was found to be statistically significant (*p* < 0.05) in Cd-administered rats compared to a non-significant increase recorded with Cd-co-administered rats with vitamin C, zinc or NAC either individually or in combination which fell in the range of 237 to 238 g. The body weight gain was found to be approximately 5% and was not significant in Cd-co-administered with vitamin C, zinc or NAC either individually or in combinations.

The individual liver weights of rats of different experimental batches showed a significant decrease (*p* < 0.05) ranging between 23 to 56%. Maximum decrement (−55.54%) was recorded with Cd-treated batch of rats compared to control batch, whereas 23 to 33% was recorded with other experimental batches of rats. Histosomatic index (HSI) of liver tissue was found to be statistically significant (*p* < 0.05) and decreased in all the experimental batches of rats except Cd-treated batch of rats found to be not significant (NS). The relative weights of kidney tissue, also found to be significant (*p* < 0.05), decreased in all the experimental batches of rats. The percentage of decrease was in a range between 27 to 44% in all the experimental batches of rats. The HSI of kidneys was found to be significantly decreased (*p* < 0.05) in all the experimental batches of rats except the Cd-treated batch of rats, which was found to be not significant (NS).

### 3.2. Respiratory and Antioxidant Status

Basal metabolic rate (BMR) and tissue respiratory (TR) potentials of both liver and kidney were monitored and are presented in Table 2. The BMR and TR values were found to be significantly (*p* < 0.05) decreased in all the experimental batches of rats compared to the control batch. The biochemical parameters of serum were monitored and are presented in Table 3. The total protein, albumin, urea, creatinine, lactate dehydrogenase (LDH), aspartate amino transferase (AST) and alanine amino transferase (ALT) were found to be significantly (*p* < 0.05) elevated or increased in all the experimental batches of rats compared to the control batch of rats. The antioxidant enzymes and products estimated in liver and kidney tissues in all the experimental batches of rats are presented in Table 4 and Table 5, respectively. Superoxide dismutase (SOD), catalase (CAT), glutathione peroxidase (GPx) and glutathione (GSH) contents were found to be significantly (*p* < 0.05) decreased in all the batches of experimental rats compared to the control batch of rats. Lipid peroxidation values were significantly (*p* < 0.05) increased in all the batches of experimental rats compared to the control batch.

## 4. Discussion

Generally, in biological systems, heavy metals, including Cd, have been reported to affect cellular organelles and components, such as the cell membrane, mitochondria, lysosomes, endoplasmic reticulum and some enzymes involved in metabolism, detoxification and damage repair [24]. All heavy metals are also classified as human carcinogens (known or probable) according to the U.S. Environmental Protection Agency (USEPA), and the International Agency for Research on Cancer (IARC). The toxic heavy metals are known to create environmental issues [2]. The USEPA recommended the limit of Cd in drinking water at 0.003 to 0.005 mg/L.

### 4.1. Body Weights and Relative Organ Weights

In xenobiotic studies, it has been reported that body and organ weights are considered an important criterion for the evaluation of organ toxicity. The decrease or increase in body weights during Cd toxicity generally reflects the toxic effects of Cd or xenobiotic agents. The body weights and relative organ weights of the experimental rats, i.e., the Cd-treated batch and those co-administered with vitamin C, zinc or NAC, either individually or in combination, were significantly reduced. Immunization evokes a lot of pain, distress and inflammation, consequently reducing the animals’ movement and appetite, thereby leading to a significant decrease in food uptake, i.e., anorexia or food avoidance or post-food palatability due to Cd toxicity. Cd-induced toxicity involves the induction of oxidative stress resulting in alterations in antioxidant status, leading to severe metabolic disorders and weight loss. Several earlier reports also state that inflammation causes weight loss ranges between 1–20% due to Cd-induced toxicity or treatment [25]. In the present study, vitamin C, zinc, and NAC are considered more efficient in the attenuation of Cd-induced toxicity, restoring metabolic status, increasing food intake, body weight, organ and body weights to the maximum possible extent.

### 4.2. Respiratory and Antioxidant Status

Generally, the level of organismic metabolism is known by measuring the rate of oxygen consumption. It is known that respiratory rates are under the influence of biotic and abiotic factors. The rate of oxygen consumption of the whole animal, as well as its tissues, reflects the metabolic rate, which can be taken as an index of the metabolic status of the animal. Therefore, the measurement of O_2_ consumption can be constructed as a bio-detector of pollution of different types, including heavy metal pollution, as its toxic nature causes stress to the animal. The rate of O_2_ consumption is taken as a parameter to assess the toxic impact of Cd and provides useful information on energy metabolism. Since the present study concerns the metabolic status of rats under Cd toxicity, the study of the whole animal and tissue respiratory potentials is essential to determine metabolic status. The results obtained in the present clearly demonstrate that Cd toxicity induces a hypoxic stress condition in rats reflected in decreased O_2_ consumption at the tissue level, i.e., liver and kidney tissue. Consistent with total respiration, unit metabolism also showed a significant decrease under Cd-intoxication. This indicates that there was a shift in the metabolic emphasis from aerobiosis to anaerobiosis as a form of metabolic compensation to overcome the Cd toxicity.

In the present investigation, an attempt was made to study the toxic impact of Cd in male rats, and furthermore, we were very interested to assess potential amelioration by vitamin C, zinc and NAC co-administration with Cd, individually and in combination. It has been recognized that Cd is considered one of the most toxic components of environmental and industrial pollutants, and is assumed to induce oxidative damage. Previously, several authors reported that Cd is known specifically to induce oxidative damage in rats [3,8]. Cd administration into the biological system depends on the route of exposure, the dose administered and the duration of exposure [4]. The maximum rate of Cd accumulation was up to 70% recorded in the cytoplasm, followed by 15% in the nucleus, with relatively low quantities recorded in the endoplasmic reticulum and mitochondria [26]. Previously it was reported that Cd administration with selected chemicals or minerals or vitamin supplementation facilitated reduction in Cd-induced toxicity to the maximum possible extent [8]. In the present study, SOD, CAT and GPx activities were found to be significantly decreased, and there was decreased activity of redox cycling antioxidant enzymes and increase in lipid peroxidation in the liver and kidney tissues of rats. This effect has been associated with oxidative stress. The activities of SOD and CAT were decreased in Cd-treated rats and increased in rats administered with vitamin C, zinc, and NAC, either individually or in combination. The decreased activities of SOD and CAT may be due to a concomitant increase in the generation of free radicals in the tissues of rats, consequent upon Cd administration. The interaction between Cd and essential trace elements may be one of the reasons for the decrease in antioxidant enzymes in the tissues of rats, including the liver and kidney, because Cd can occupy the zinc site in Cu/Zn-SOD and creates inactive forms of the enzyme (Cu/Cd-SOD) [27]. However, the activity of Cu/Zn-SOD was increased in rats supplemented with vitamin C, zinc and NAC. This may have occurred because the supplements protect against the cytotoxicity of Cd, permitting the maintenance of normal cellular redox balance by blocking free radical generation.

The liver SOD activity was significantly decreased, which may be attributed to lipid peroxyl radicals and inactivation of their breakdown products. It has already been established that increased MDA levels were found to inhibit SOD activity levels significantly, supporting this hypothesis. Several authors have reported that significant increases in protein oxidation levels and lipid peroxides in individuals with known chronic liver disease conditions are induced by hepato-toxic xenobiotics [28]. GPx activity levels were significantly decreased (*p* < 0.05) during Cd-intoxication, which may be attributed to the enhancement of peroxide damage to PUFA, consequently leading to maximum levels of lipid peroxidation at tissue level or cumulative accumulation of ROS also leading to tissue damage. This observation implies that free radicals are involved in tissue damage during Cd-intoxication [29]. It has been observed that Cd significantly reduced the catalytic efficiency of GPx directly, which subsequently led indirectly to reduced concentrations of glutathione and NADPH necessary for effective activity [30]. However, changes in GPx activity caused by Cd toxicity was ameliorated by supplementation with co-administration of vitamin C, zinc and NAC which enhance the anti-oxidative defense system, thereby providing protection against Cd toxicity [31].

Cd-intoxication significantly elevated the LPO concentrations in the tissues of rats, thereby causing the initiation of lipid peroxidation processes at tissue level. This suggests that Cd may induce oxidative stress via the production of hydroxyl radicals, superoxide ions, nitric oxide and H_2_O_2_ [7,32]. The activity of GPx was significantly decreased in rats co-supplemented with vitamin C, zinc and NAC individually and in combination due to the effects of the antioxidant defense system which protects cells from Cd-induced toxicity. Supplementation is known to play a vital role in the maintenance of GPx concentrations and in protecting the integrity and function of tissues [10,33]. It is acknowledged that hepatic GSH acts as an electrophile, radical scavenger and redox partner. GSH may also serve as a co-factor for several drug-metabolizing enzymes, i.e., GSTs, which are consumed or for antioxidant enzymes, i.e., GPx, where it serves as a redox partner [34]. During oxidation, GSH forms a dimer, glutathione disulfide (GSSG), which, in turn, can be reduced by the enzyme glutathione reductase at the expense of NADPH [34].

The reduction in the activities of antioxidant enzymes in our study is consistent with several earlier studies, which also reported the same disorders [3,25]. Organisms may have an endogenous protective antioxidant defense system against damage by free oxygen radicals. SOD, CAT and GPx are enzymatic antioxidants that catalyze detoxification reactions of toxic oxygen metabolites. The failure of the primary antioxidant system to act against free radicals generated may reflect the inability of liver mitochondria and microsomes to eliminate H_2_O_2_ produced after exposure to Cd. This may also be due to enzyme inactivation caused by excess ROS production in mitochondria and microsomes [35]. All these findings are confirmed through histopathological studies (unpublished data), showing congestion of hepatoportal blood vessels, inflammatory infiltrations, and degeneration of hepatocytes with necrotic foci scattered throughout the liver. Several experimental studies have, though, shown that Cd is relatively rapidly metabolized and excreted in mammalian models, including rats [3,36]. It was reported that an accumulation of Cd in several target organs, including liver, gonads, brain, etc., caused damage at a cellular level leading to disturbances in several metabolic and biochemical pathways following exposure to different doses of Cd [3,9].

From the results obtained, we understand that Cd toxicity induced damage that occurred in liver and kidney tissues, as observed through pathological studies. Both the liver and kidney are considered important organs for metabolic processes, including detoxification, storage and excretion of xenobiotics and their metabolites. The physiological and biochemical functions associated with the liver and kidney implies that they are especially vulnerable to damage. In the present study, an attempt was made to assess the impact of Cd toxicity followed by its amelioration with co-administration of vitamin C, zinc and NAC, both individually and in combination in rats. Serum enzymes, including AST, ALT, LDH and biomolecules, such as albumin, urea and creatinine were mainly considered as biomarkers for the evaluation of hepatic damage under Cd toxicity. In the present study, Cd toxicity caused significant elevation in the activity levels of several serum enzymes, such as, AST, ALT and LDH. In contrast, ALP activity showed a significant decrease in activity compared to the control batch. In addition to the above, increased levels of hepatic serum markers also indicated extensive damage to the liver tissue during Cd toxicity. LPO is one of the main manifestations of oxidative damage which plays an important role in the toxicity of several xenobiotics [9].

Moreover, it has been confirmed that Cd toxicity caused a significant increase in lipid peroxidation concentrations in the liver tissue of rats, since it causes LPO in numerous tissues, both in vivo and in vitro [36]. Cd toxicity is likely to induce oxidative stress by the production of hydroxyl radicals, superoxide anions, nitric oxide and H_2_O_2_ [9]. Moreover Cd treatment also causes both structural and functional damage to the cell membrane, significantly increasing permeability, thereby resulting in leakage of hepatic enzymes into the blood. Furthermore, liver damage was also confirmed during Cd toxicity with a significant increase in the levels of plasma components, including albumin, bilirubin etc.

Therefore, the increased activities of ALT and AST in plasma can be attributed to the leakage of these enzymes from the liver cytosol into the bloodstream. Earlier reports have suggested that lysosomal instability caused by Cd toxicity resulted in the leakage of hepatic enzymes, including ALT, AST and ALP into the blood stream [37]. Consonant with the above observations, there was a significant increased activity level recorded for AST and ALT; changes in ALT activities may also be attributed to the significant damage occurred during Cd toxicity. The changes in ALP activities may also have contributed to cholestasis and acute hepatocellular necrosis. Earlier reports suggest that Cd toxicity causes significant elevation in liver enzymes, including SGOT, SGPT and ALP, clearly indicating liver dysfunction. In biological systems, among the tissues the kidneys play a vital role not only in the elimination of several metabolites and unmodified drugs but also in terms of performing biotransformation reactions. From the results obtained in the present study, it was established that Cd intoxication induced nephrotoxicity, leading to the accumulation of several metabolites in this tissue, subsequently leading to kidney damage and ultimately leading to the death of the organism. Earlier reports are also available concerning Cd effects on the kidney [7].

The effects of Cd toxicity in the liver and kidney, and the pathology related to both nephrotoxicity and hepatotoxicity, requires further in-depth investigation, to answer several questions. The tissues have higher affinity against Cd molecules and they contain high concentrations of cytochrome P450. Several authors have reported that Cd toxicity disrupts the structural and functional organization of both liver and kidney^1^. LPO is a lipid peroxidation marker. Several earlier studies quantified the levels of MDA to assess and monitor oxidative stress as an indicator of damage of lipid components, but the LPO process was selected in the present study for accurate measurement of oxidative damage. The increase in LPO level in the Cd-exposed liver and kidney tissues compared to the control batch indicates not only the intensification of lipid peroxidation but also a significant increase in oxidative stress.

An important indicator of oxidative cellular damage is also the presence of H_2_O_2_ which is a natural product of cellular metabolism Because it possesses relatively strong properties, H_2_O_2_ is highly reactive and found to be a potent toxic substance. Under normal physiological conditions, H_2_O_2_ is deactivated by CAT and GPx. Generally, under normal physiological conditions, H_2_O_2_ molecules will be deactivated by certain enzymes, such as CAT and GPx, which prevent excessive accumulation of H_2_O_2_ in the cells, thereby protecting the organism from the destructive effects on several biomolecules at a cellular level. The available study outcomes suggest that antioxidants, such as vitamins, polyphenyls, and metals may protect the organism exposed to Cd toxicity against oxidative stress. It has been observed that both urea and creatinine from serum were found to be significantly elevated during Cd intoxication which can be attributed to an imbalance in the oxidative metabolism in tissues such as the liver and kidney, which correlates with previous reports [38], where creatinine and urea levels significantly increased during Cd toxicity. The significantly elevated level of creatinine during Cd toxicity may be attributed to the oxidative damage to tissues such as the liver and kidney. Deterioration of the tissue architecture during Cd toxicity will lead to the release of creatinine into the blood with such situations with Cd toxicity reported by earlier authors [38]. Tissue damage was also linked to defects in infiltration. Earlier reports suggest that increase in creatinine levels is an indication of renal tubular damage due to Cd-induced nephrotoxicity [4,38].

In the present study, an attempt was also made to monitor the rate of amelioration of Cd toxicity by co-administration of vitamin C, zinc and NAC in rat tissues, such as the liver and kidney. A very effective therapeutic method, commonly used for heavy metal poisoning, is chelation therapy, which promotes the excretion of metals. Natural antioxidant compounds include vitamins C and E, which are good chelators used in metal toxicity management. Vitamin C (ascorbic acid) is an important dietary antioxidant which significantly decreases the adverse effects of reactive species, including reactive oxygen and nitrogen species known to cause extensive damage to several macromolecules, such as lipids, proteins and DNA, which are implicated in chronic diseases, including cardiovascular disease, stroke, cancer, neurodegenerative diseases and cataractogenesis. Ascorbic acid is a potent, water-soluble antioxidant capable of scavenging or neutralizing an array of ROS, including hydroxyl, alkolyl, peroxyl, superoxide anion, hydroperoxyl radicals and reactive nitrogen radicals, such as nitrogen dioxide, nitroxide, and peroxynitrile at very low concentrations [39,40].

Vitamin C is an essential co-factor for many enzymes involved in diverse metabolic pathways. In the present study co-administration of vitamin C significantly ameliorated Cd toxicity in rat tissues which were monitored through activity levels of antioxidant enzymes in tissues and in serum. Previous reports suggest that vitamin C significantly protected against Cd toxicity, i.e., activity levels of ALT, AST, SOD, CAT, SGOT, and SGPT were significantly altered in Cd-induced toxicity in rats and rabbits [40,41,42].

It has been well established that antioxidants present at the tissue level play a vital role in the performance of antioxidant defense mechanisms to overcome oxidative impairment, thereby protecting biological cell functions [43]. Among the range of antioxidants, zinc plays an important role in the regulation of the antioxidant system [38,44]. Zinc interacts with heavy metals, such as Cd, in metabolism and toxicity [45]. Zinc neutralizes the toxic effect of Cd in many tissues, in particular, preventing kidney damage. The results obtained in the present study clearly indicate the protective nature of zinc in Cd toxicity in rat tissues. Many studies have indicated that zinc has protective effects on Cd-induced changes of oxidative enzymes, such as SOD, CAT, GPx etc. Moreover, competitive absorption of Cd and zinc can influence Cd susceptibility. Enhanced daily zinc intake has been suggested to protect the organism against Cd accumulation and subsequent toxicity. In one of the experimental batches, NAC was co-administered with Cd to monitor the amelioration potential in rat tissues. NAC administration was shown to be effective in reducing oxidative impairment in tissues such as the liver and kidney of rats, efficiently increasing antioxidant status.

The present study has demonstrated that cadmium is a toxic element that causes damage to the liver and kidney tissues of rats. Vitamin C, zinc and NAC have been shown to have protective roles against Cd toxic effects. The results obtained in the present study pertaining to basal metabolic rate, tissue respiratory potentials, antioxidant enzyme activity levels, and serum biomarker enzymes and antioxidant product quantities, clearly demonstrate that all the antioxidant agents selected in the present study, vitamin C, zinc and NAC, had nearly the same protective effects against Cd toxicity at the tissue level. The co-administration of vitamin C, zinc and NAC with Cd, suggests beneficial effects in the amelioration of Cd toxicity. Further studies are necessary to understand other possible protective effects of the above selected agents in subjects exposed to Cd and to establish the exact pathways or reactions at the cellular level.

## 5. Conclusions

The results obtained in the present study clearly demonstrate, using specific biomarkers for monitoring Cd toxicity, that co-administration of vitamin C, zinc and NAC ameliorates the effects of Cd toxicity in rats.

## Figures and Tables

**Table 1:** Body and relative organ weight in control and experimental rats.

Parameters	Control	Cd Treated	Vit-C	Zinc	NAC	C + Zinc	C + NAC	Zinc + NAC	C + Zinc +NAC
IBW	225.19 ±10.12	226.12 ±10.14	225.18 ±9.79	224.19 ±10.17	225.49 ±10.15	225.42 ±10.19	226.74 ±10.45	225.48 ±10.12	224.14 ±10.15
FBW	268.15 ^a^ ±10.17	122.35 ^b^ ±10.22	238.14 ^b,c^ ±10.12	237.19 ^b,c^ ±10.03	238.32 ^b,c^ ±10.05	238.05 ^b,c^ ±10.14	237.78 ^b,c^ ±9.89	238.04 ^b,c^ ±9.88	238.05 ^b,c^ ±10.13
BWG/L	+19.07 ^b^	−45.89 ^a^	+5.76 ^b^	+5.79 ^b^	+5.69 ^b^	+5.60 ^b^	+4.87 ^b^	+5.57 ^b^	+5.60 ^b^
LW	10.84 ^a^ ±0.63 PDC	4.82 ^b,c^ ±0.25 −55.54	8.12 ^b,c^ ±0.45 −25.09	8.24 ^b,c^ ±0.38 −23.99	8.25 ^b,c^ ±0.39 −23.89	8.18 ^b,c^ ±0.42 −32.52	8.25 ^b,c^ ±0.41 −23.89	8.05 ^b,c^ ±0.38 −27.74	8.19 ^b,c^ ±0.35 −24.45
HIS-L	4.04 ^a^ PDC	3.94 ^a^ −2.48	3.41 ^b^ −15.59	3.48 ^b^ −13.86	3.46 ^b^ −14.36	3.44 ^b^ −14.85	3.47 ^b^ −14.11	3.38 ^b^ −16.34	3.44 ^b^ −14.85
KW	2.93 ^a^ ±0.28 PDC	1.23 ^b^ ±0.14 −44.37	2.08 ^b,c^ ±0.15 −29.01	2.12 ^b,c^ ±0.17 −27.65	2.11 ^b,c^ ±0.14 −27.99	2.14 ^b,c^ ±0.17 −29.96	2.14 ^b,c^ ±0.18 −26.96	2.12 ^b,c^ ±0.15 −27.65	2.11 ^b,c^ ±0.15 −27.99
HIS-K	1.09 ^a^ PDC	1.01 ^a^ −7.34	0.87 ^b^ −20.18	0.89 ^b^ −18.35	0.89 ^b^ −18.35	0.90 ^b^ −17.43	0.90 ^b^ −17.43	0.89 ^b^ −18.35	0.89 ^b^ −18.35

Values are expressed as mean ± SD of six individual observations. ^a,b,c^ Values not sharing a common superscript letter (^a,b,c^) differ significantly at *p* < 0.05 (DMRT). PDC, percent deviation over control; IBW, initial body weight (g); FBW, final body weight (g); BWG/L (%), body weight gain/loss g in 45 days; LW, liver weight (g); HIS-L, histosomatic index of liver; KW, kidney weight (g); HIS-K: histosomatic index of kidney.

**Table 2:** Basal Metabolic Rate (BMR) and Tissue Respiration in Selected tissues in Control and Experimental rats.

Parameters	Control	Cd Treated	Vit-C	Zinc	NAC	C + Zinc	C + NAC	Zinc + NAC	C + Zinc +NAC
Basal Metabolic Rate (BMR)	4.23 ^a^ ±0.24 PDC	2.72 ^b^ ±0.13 −35.70	3.41 ^b,c^ ±0.13 −19.39	3.54 ^b,c^ ±0.15 −16.31	3.53 ^b,c^ ±0.16 −16.55	3.54 ^b,c^ ±0.13 −16.31	3.48 ^b,c^ ±0.15 −17.73	3.53 ^b,c^ ±0.14 −16.55	3.54 ^b,c^ ±0.15 −16.31
Tissue Respiration (TR), Liver	1033.46 ^a^ ±24.77 PDC	642.14 ^b^ ±18.15 −37.87	842.18 ^b,c^ ±16.19 −18.51	852.14 ^b,c^ ±17.15 −17.55	860.15 ^b,c^ ±17.19 −16.77	862.19 ^b,c^ ±17.14 −16.57	863.14 ^b,c^ ±16.75 −16.48	861.15 ^b,c^ ±17.13 −16.67	867.18 ^b,c^ ±14.19 −16.09
Tissue Respiration (TR), Kidney	858.77 ^a^ ±12.85 PDC	508.42 ^b^ ±12.12 −40.80	660.18 ^b,c^ ±12.19 −23.12	672.13 ^b,c^ ±15.14 −21.73	673.13 ^b,c^ ±11.14 −21.61	671.72 ^b,c^ ±13.49 −21.78	675.70 ^b,c^ ±14.19 −21.32	673.49 ^b,c^ ±13.45 −21.58	677.43 ^b,c^ ±14.46 −21.12

Values are expressed as mean ± SD of six individual observations. ^a,b,c^ Values not sharing a common superscript letter (^a,b,c^) differ significantly at *p* < 0.05 (DMRT). PDC, percent deviation over control; BMR, mL of oxygen consumed/G weight of animal/h; TR, µL of oxygen consumed/G weight of tissue/h.

**Table 3:** Biochemical Parameters in Tissues/Serum of Rats under different Experimental treatments.

Parameters	Control	Cd Treated	Vit-C	Zinc	NAC	C + Zinc	C + NAC	Zinc + NAC	C + Zinc + NAC
Total Protein (mg/mL)	15.38 ^a^ ±1.29 PDC	33.49 ^b^ ±2.28 +117.75	20.31 ^b,c^ ±1.72 +32.05	25.34 ^b,c^ ±1.83 +64.48	26.77 ^b,c^ ±1.49 +74.06	26.82 ^b,c^ ±1.34 +74.97	22.14 ^b,c^ ±1.13 +43.95	20.74 ^b,c^ ±1.14 +34.85	20.18 ^b,c^ ±1.12 +31.21
Albumin (mg/mL)	2.38 ^a^ ±0.22 PDC	2.89 ^b^ ±0.23 +21.43	3.04 ^b,c^ ±0.23 +27.73	3.08 ^b,c^ ±0.24 +29.41	3.12 ^b,c^ ±0.25 +31.09	2.94 ^b,c^ ±0.22 +23.53	2.95 ^b,c^ ±0.22 +23.95	2.99 ^b,c^ ±0.24 +25.63	3.02 ^b,c^ ±0.22 +26.89
Urea (mg/dL)	34.14 ^a^ ±2.12	41.19 ^b^ ±2.74	38.77 ^b,c^ ±2.49	39.42 ^b,c^ ±2.49	38.45 ^b,c^ ±2.44	39.25 ^b,c^ ±2.38	37.77 ^b,c^ ±2.74	38.41 ±2.49	36.77 ^b,c^ ±1.42
Creatinine (mg/dL)	0.65 ^a^ ±0.04 PDC	0.83 ^b^ ±0.08 + 20.62	0.82 ^b,c^ ±0.07 +13.56	0.79 ^b,c^ ±0.08 +21.54	0.78 ^b,c^ ±0.07 +20.00	0.77 ^b,c^ ±0.07 +18.46	0.74 ^b,c^ ±0.08 +13.85	0.73 ^b,c^ ±0.08 +12.31	0.74 ^b,c^ ±0.07 +13.85
LDH (µ moles of formazon formed/mg protein/h)	18.14 ^a^ ±0.25 PDC	39.79 ^b^ ±0.34 +119.35	35.14 ^b,c^ ±0.29 +93.72	34.72 ^b,c^ ±0.27 +91.40	27.45 ^b,c^ ±0.25 +51.32	25.79 ^b,c^ ±0.34 +42.18	24.74 ^b,c^ ±0.31 +36.38	21.74 ^b,c^ ±0.31 +19.85	25.78 ^b,c^ ±0.29 +42.12
AST (U/L)	53.14 ^a^ ±2.77 PDC	459.78 ^b^ ±25.85 +765	254.72 ^b,c^ ±15.75 +379	277.45 ^b,c^ ±18.74 +422	245.42 ^b,c^ ±19.45 +361	205.74 ^b,c^ ±18.42 +287	214.12 ^b,c^ ±20.14 +303	205.74 ^b,c^ ±18.85 +287	201.71 ^b,c^ ±18.45 +279
ALT (U/L)	39.77 ^a^ ±2.04 PDC	365.72 ^b^ ±20.14 +196	204.14 ^b,c^ ±15.05 +413	201.45 ^b,c^ ±19.41 +407	194.15 ^b,c^ ±19.42 +388	190.45 ^b,c^ ±20.42 +379	185.45 ^b,c^ ±20.14 +366	168.74 ^b,c^ ±14.55 +324	158.74 ^b,c^ ±12.85 +299
ALP (U/L)	108.12 ^a^ ±8.14 PDC	38.19 ^b^ ±2.15 −64.68	62.13 ^b,c^ ±3.75 −42.54	68.34 ^b,c^ ±3.49 −36.79	70.13 ^b,c^ ±3.74 −35.14	73.14 ^b,c^ ±4.18 −32.35	75.18 ^b,c^ ±4.42 −30.47	78.11 ^b,c^ ±4.49 −27.76	80.13 ^b,c^ ±4.13 −25.87

Values are expressed as mean ± SD of six individual observations. ^a,b,c^ Values not sharing a common superscript letter (^a,b,c^) differ significantly at (*p* < 0.05 (DMRT)). PDC, percent deviation over control.

**Table 4:** Levels of Antioxidant Enzymes and Products in Liver tissue of Control and Experimental rats.

Parameters	Control	Cd Treated	Vit-C	Zinc	NAC	C + Zinc	C + NAC	Zinc + NAC	C + Zinc + NAC
MDA	475 ^a^ ±25 PDC	1015 ^b^ ±39 +114	885 ^b,c^ ±29 +86	901 ^b,c^ ±40 +90	950 ^b,c^ ±32 +100	809 ^b,c^ ±29 +70	804 ^b,c^ ±32 +69	775 ^b,c^ ±29 +64	670 ^b,c^ ±32 +41
SOD	66.79 ^a^ ±2.15 PDC	31.73 ^b^ ±1.14 −35	34.44 ^b,c^ ±1.18 −48	36.18 ^b,c^ ±1.15 −46	38.14 ^b,c^ ±1.19 −43	40.12 ^b,c^ ±1.28 −40	42.14 ^b,c^ ±1.29 −37	44.75 ^b,c^ ±1.32 −33	51.14 ^b,c^ ±1.12 −23
CAT	88.14 ^a^ ±2.12 PDC	42.44 ^b^ ±1.13 −52	44.75 ^b,c^ ±1.14 −49	47.75 ^b,c^ ±1.25 −46	49.75 ^b,c^ ±1.32 −43	53.72 ^b,c^ ±1.34 −40	59.14 ^b,c^ ±1.42 −37	60.19 ^b,c^ ±1.38 −33	68.13 ^b,c^ ±1.18 −23
LPO	5.14 ^a^ ±0.28 PDC	9.85 ^b^ ±0.38 +92	8.35 ^b,c^ ±0.32 +63	8.19 ^b,c^ ±0.31 +59	7.74 ^b,c^ ±0.26 +51	7.15 ^b,c^ ±0.25 +39	6.74 ^b,c^ ±0.27 +31	6.34 ^b,c^ ±0.26 +23	6.15 ^b,c^ ±0.24 +20
GP_X_	8.15 ^a^ ±0.46 PDC	4.05 ^b^ ±0.28 −50	4.18 ^b,c^ ±0.24 −49	4.34 ^b,c^ ±0.25 −47	4.75 ^b,c^ ±0.26 −42	5.25 ^b,c^ ±0.29 −36	5.79 ^b,c^ ±0.32 −29	6.42 ^b,c^ ±0.35 −22	7.04 ^b,c^ ±0.28 −14
GSH	4.93 ^a^ ±0.41 PDC	2.85 ^b^ ±0.22 −42	2.92 ^b,c^ ±0.23 −41	2.94 ^b,c^ ±0.24 −40	3.02 ^b,c^ ±0.27 −39	3.14 ^b,c^ ±0.29 −36	3.25 ^b,c^ ±0.32 −34	3.32 ^b,c^ ±0.33 −33	3.49 ^b,c^ ±0.32 −29

Values are expressed as mean ± SD of six individual observations. ^a,b,c^ Values not sharing a common superscript letter (^a,b,c^) differ significantly at *p* < 0.05 (DMRT). PDC, percent deviation over control; MDA, µ moles/g wet wt of tissue; SOD, superoxide dismutase units/g wet weight of tissue/minute; CAT, catalase activity µ moles of H_2_O_2_ degladed/g wet wt of tissue/minute; LPO, µ moles of maloaldehyde formed/g wet weight; GP_X_, glutathione peroxidase nanomoles of NADPH oxidized/mg protein/minute; GSH, glutathione nanomoles/g wet weight of tissue.

**Table 5:** Levels of Antioxidant Enzymes and Products in Kidney tissue of Control and Experimental rats.

Parameters	Control	Cd Treated	Vit-C	Zinc	NAC	C + Zinc	C + NAC	Zinc + NAC	C + Zinc + NAC
MDA	528 ^a^ ±29 PDC	1114 ^b^ ±35 +111	1074 ^b,c^ ±38 +103	995 ^b,c^ ±24 +88	880 ^b,c^ ±22 +67	740 ^b,c^ ±25 +40	730 ^b,c^ ±26 +38	722 ^b,c^ ±27 +37	685 ^b,c^ ±24 +30
SOD	45.72 ^a^ ±2.14 PDC	24.12 ^b^ ±1.10 −47	26.19 ^b^ ±1.12 −43	28.12 ^b^ ±1.14 −38	30.12 ^b,c^ ±1.15 −34	31.18 ^b,c^ ±1.14 −32	32.18 ^b,c^ ±1.14 −30	33.11 ^b,c^ ±1.14 −28	33.75 ^b,c^ ±1.71 −26
CAT	82.18 ^a^ ±2.12 PDC	43.42 ^b^ ±1.14 −47	45.45 ^b^ ±1.13 −45	48.74 ^b^ ±1.14 −41	51.23 ^b^ ±1.24 −38	53.42 ^b,c^ ±1.42 −35	58.41 ^b,c^ ±1.56 −29	60.79 ^b,c^ ±1.57 −26	65.77 ^b,c^ ±2.12 −20
LPO	4.73 ^a^ ±0.24 PDC	8.73 ^b^ ±0.42 +85	8.14 ^b,c^ ±0.33 +72	8.04 ^b,c^ ±0.32 +70	7.34 ^b,c^ ±0.29 +55	7.49 ^b,c^ ±0.28 +58	6.43 ^b,c^ ±0.31 +36	6.05 ^b,c^ ±0.25 +28	5.79 ^b,c^ ±0.23 +22
GP_X_	7.32 ^a^ ±0.38 PDC	3.92 ^b^ ±0.24 −46	4.14 ^b^ ±0.24 −43	4.54 ^b^ ±0.25 −38	4.89 ^b,c^ ±0.26 −33	5.12 ^b,c^ ±0.26 −30	5.34 ^b,c^ ±0.25 −27	5.59 ^b,c^ ±0.24 −24	6.13 ^b,c^ ±0.25 −16
GSH	4.12 ^a^ ±0.34 PDC	2.42 ^b^ ±0.22 −41	2.63 ^b,c^ ±0.22 −36	2.79 ^b,c^ ±0.21 −32	2.84 ^b,c^ ±0.23 −31	3.02 ^b,c^ ±0.23 −27	3.12 ^b,c^ ±0.24 −24	3.23 ^b,c^ ±0.24 −22	3.38 ^b,c^ ±0.24 −18

Values are expressed as mean ± SD of six individual observations. ^a,b,c^ Values not sharing a common superscript letter (^a,b,c^) differ significantly at *p* < 0.05 (DMRT). PDC: percept deviation over control; MDA, µ moles/g wet wt of tissue; SOD, superoxide dismutase units/g wet weight of tissue/minute; CAT, catalase activity µ moles of H_2_O_2_ degladed/g wet wt of tissue/minute; LPO, µ moles of maloaldehyde formed/g wet weight; GP_X_, glutathione peroxidase nanomoles of NADPH oxidized/mg protein/minute; GSH, glutathione nanomoles/g wet weight of tissue.

## Data Availability

Data generated during this research study are available upon request.
